# Supplementary material for: Heroin Self-Administration and Extinction Increase Prelimbic Cortical Astrocyte–Synapse Proximity and Alter Dendritic Spine Morphometrics That Are Reversed by N-Acetylcysteine
Source: Cells. 2023 Jul 8;12(14):1812. doi: 10.3390/cells12141812 (PMC10378353; doi:10.3390/cells12141812)
Supplement: Supplementary file 1 [file cells-12-01812-s001.zip › cells-2255586-supplementary.pdf]

Virus	Description/Use	Source
AAV5.*GfaABC1D*::tdTomato	*GfaABC1D* promoter driven expression of tdTomato.	Addgene #44332-AAV5
AAV5.*GfaABC1D*::LcK-EGFP	*GfaABC1D* promoter driven insertion of EGFP into astrocyte plasmalemma to label astrocytes	Addgene #105598-AAV5
AAV1.*CAG*.Flex::Ruby2sm-FLAG	Cre-recombinase dependent CAG promoter driven delivery of FLAG tagged non-fluorescent variant of mRuby2 to label neurons for dendritic spine analysis	Addgene #98928-AAV1
rgAAV.*hSyn*::Cre-WPRE.hGH	Human synapsin promoter driven retrograde delivery of cre-recombinase to neurons	Addgene #105553-AAVrg
AAV5.*GfaABC1D*::shTSP2-GFP	*GfaABC1D* promoter driven expression of GFP, with a custom microRNA targeting TSP2 present in the 3’ UTR of GFP.	Gift from the laboratory of Dr. Yan Dong

Table S1: Names, descriptions and sourcing information for all AAV constructs used in these studies.
